# Taxonomic and functional trait-based approaches suggest that aerobic and anaerobic soil microorganisms allow the natural attenuation of oil from natural seeps

**DOI:** 10.1038/s41598-022-10850-4

**Published:** 2022-05-04

**Authors:** Aurélie Cébron, Adrien Borreca, Thierry Beguiristain, Coralie Biache, Pierre Faure

**Affiliations:** Université de Lorraine, CNRS, LIEC, 54000 Nancy, France

**Keywords:** Environmental microbiology, Environmental sciences

## Abstract

Natural attenuation, involving microbial adaptation, helps mitigating the effect of oil contamination of surface soils. We hypothesized that in soils under fluctuating conditions and receiving oil from seeps, aerobic and anaerobic bacteria as well as fungi could coexist to efficiently degrade hydrocarbons and prevent the spread of pollution. Microbial community diversity was studied in soil longitudinal and depth gradients contaminated with petroleum seeps for at least a century. Hydrocarbon contamination was high just next to the petroleum seeps but this level drastically lowered from 2 m distance and beyond. Fungal abundance and alpha-diversity indices were constant along the gradients. Bacterial abundance was constant but alpha-diversity indices were lower next to the oil seeps. Hydrocarbon contamination was the main driver of microbial community assemblage. 281 bacterial OTUs were identified as indicator taxa, tolerant to hydrocarbon, potentially involved in hydrocarbon-degradation or benefiting from the degradation by-products. These taxa belonging to lineages of aerobic and anaerobic bacteria, have specific functional traits indicating the development of a complex community adapted to the biodegradation of petroleum hydrocarbons and to fluctuating conditions. Fungi are less impacted by oil contamination but few taxa should contribute to the metabolic complementary within the microbial consortia forming an efficient barrier against petroleum dissemination.

## Introduction

The expansion of the oil industry in the first half of the twentieth century left behind sites where oil continues to seep to the surface. Contamination of surface soils by oil from leakage of old petroleum pumping wells or natural petroleum seeps is not necessarily a major problem because natural attenuation often limits the extent of the damage. During natural attenuation process, indigenous microorganisms degrade organic contaminants without external inputs that can promote bioremediation^1^. These natural phenomena occur in environments contaminated by many different compounds such as polychlorobiphenyls^2^ or hydrocarbons^3,4^. Natural attenuation reflects the fact that biota can adapt over time to resist contamination and efficiently degrade it in various environments and conditions. Most of the reported studies on microbiology of sites contaminated with hydrocarbons are focused on the short-term effect of the crude oil after a major contamination event (oil spill, oil pipe damage)^4–7^, but very few have studied the long-term effect of chronic crude oil seeps on the composition of microbial communities. Studies focusing on petroleum-contaminated soils in oil exploitation area have mostly tended to isolate microbes with hydrocarbon degradation properties for bioremediation application^8^. But there has been little in the way of attempts to analyse the composition of microbial communities in surface soils^9^, such as those contaminated for decades by oil seeps, to identify who are the best-adapted microbes and tend to found taxa as bioindicator of efficient petroleum degradation.

A wide diversity of microorganisms (fungi, bacteria and algae) are able to degrade and use petroleum (aliphatic and aromatic hydrocarbons) as sole carbon source using specific enzymatic machinery^10,11^. Although aerobic hydrocarbon biodegradation^11,12^ is the most energy-efficient respiration process^13^, the contaminated subsurface environments are often naturally anaerobic^14^, especially within oil plumes where the available oxygen has been depleted. Anaerobic bacterial degradation of petroleum-related compounds such as benzene^15^, toluene^16^, polycyclic aromatic hydrocarbons^17^ and alkanes^18^ has been demonstrated in aquifers, sediment and soil environments. Local consumption of oxygen^19^ or fluctuating conditions^20^ could create aerobic and anaerobic microniches in soil, then a multitude of degradation processes could coexist at a small scale, involving aerobic and anaerobic microbial functional populations^21^. Fungi could also be key players in degrading recalcitrant organic contaminants because of their variety of extracellular enzymes^22^. The fungal mobilization and degradation of contaminants could contribute to the release of bioavailable intermediates that could be further degraded by bacteria^23,24^. Hence, catabolic interactions among different microbial groups during biodegradation are extremely important. In this context, we therefore hypothesized that in environments under fluctuating conditions and receiving constant input of petroleum, both bacteria and fungi and, among bacteria, both aerobic and anaerobic taxa could coexist and be involved in hydrocarbon degradation processes. It is therefore essential to identify the whole microbial community adapted to chronic soil petroleum contamination and to estimate how far hydrocarbon contamination and its impact on communities extend around oil seeps.

In this study, we present a detailed spatial analysis of the microbial community structure in two forest soils which have both been impacted by petroleum seeps for at least a century but with contrasting soil characteristics. For both sites, we studied the hydrocarbon contamination longitudinal gradients away from oil seeps. Bacterial and fungal community composition was studied through high throughput sequencing of 16S and 18S rDNA and indicator taxa developing mainly next to the oil seeps and potential hydrocarbon-degraders were identified thanks to an original analysis rarely used in microbial ecology (TITAN) but well-adapted to environmental gradients.

## Material and methods

### Soil sampling

**Figure 1:**
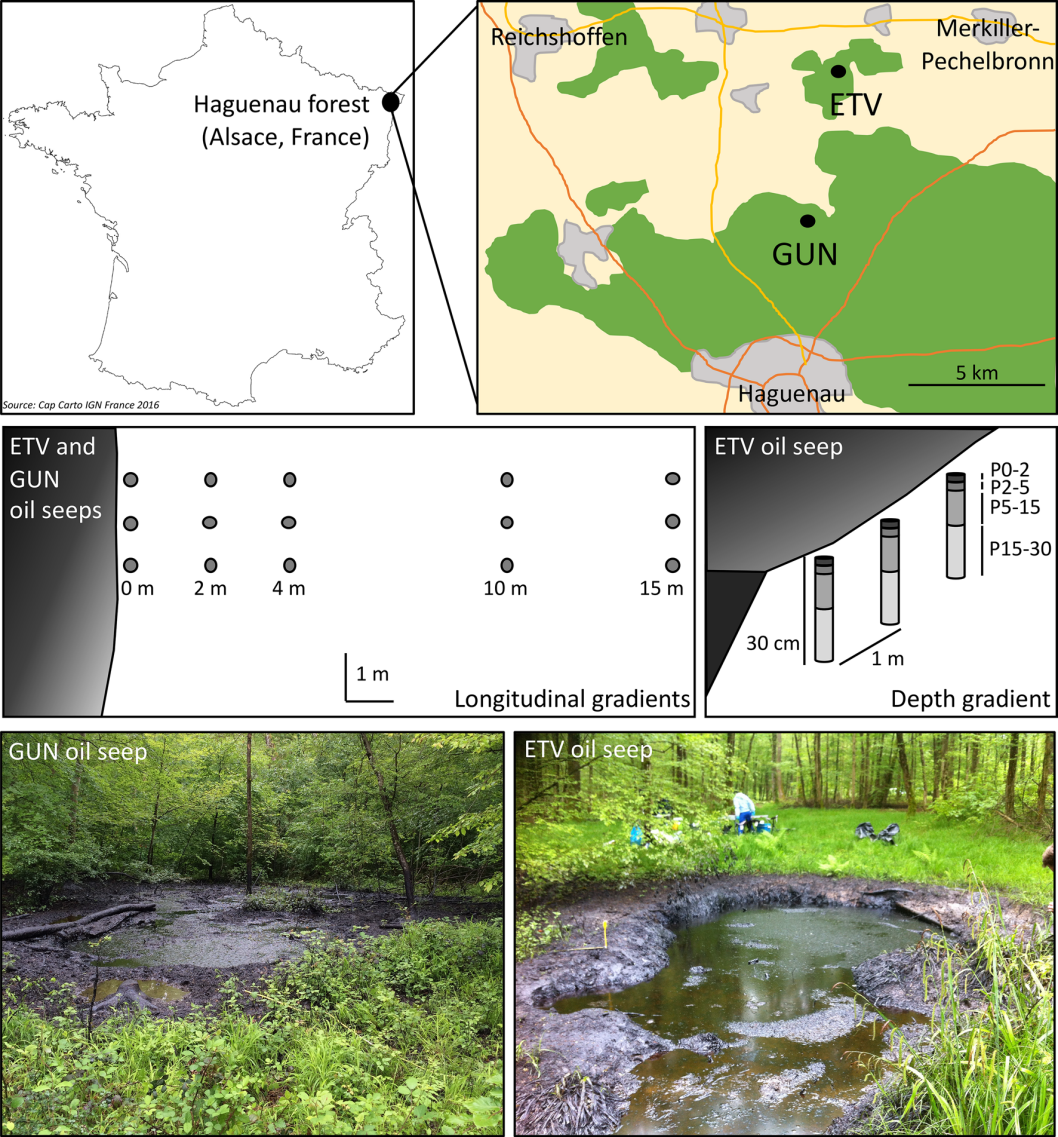
Schematic map showing the location of the ETV and GUN study sites in the Haguenau forest (France), illustrations of the sampling designs for longitudinal and depth gradients and pictures (credit P. Faure) of the ponds created by oil seeps.

The first sampling campaign was carried out in May 2016, on two sites with contrasting soil characteristics located in the Haguenau forest (Alsace, France; Fig. 1). Both sites have been impacted by oil seeps since the end of petroleum extraction which took place in the region between the end of the eighteenth century and the middle of the twentieth century by Pechelbronn petroleum. The soil of the Gunstett site (GUN; 48° 55′ 9.8" N, 7° 48′ 6.7" E) presented a clay texture (42% clay, 40% silt, 18% sand) while that of Etangs-Verts (ETV; 48° 52′ 21.8″ N, 7° 46′ 51.6″ E) is sandy loam (15% clay, 23% silt, 62% sand). This difference was also highlighted by the different cationic exchange capacities (CEC) which were of 24.5 and 6.0 cmol kg^−1^ on average in GUN and ETV soils, respectively. Continuous oil seeps created ponds of 5–10 m of diameter (Fig. 1). On each site, 15 m longitudinal transects from each oil seep were carried out and 5 sampling zones were chosen at 0, 2, 4, 10 and 15 m from the oil seeps (named GUN-0, GUN-2, GUN-4, GUN-10, GUN-15 and ETV-0, ETV-2, ETV-4, ETV-10, ETV-15). Surface core soil samples (0 to 5 cm deep) were taken in triplicates (3 subsites 1 m apart from each other) in the 5 sampling zones, representing 30 samples (2 sites × 5 sampling zones × 3 replicates; Fig. 1).

The second sampling campaign focused only on the Etangs-Verts (ETV) site and was carried out in May 2017. Only the first zone (0 m, ETV-0), the closest to the oil seep, was sampled (Fig. 1). Three independent soil cores of 30 cm depth were taken and were separated into 4 samples, to recover samples from 0–2 cm, 2–5 cm, 5–15 cm and 15–30 cm deep (named ETV0_P0-2, ETV0_P2-5, ETV0_P5-15 and ETV0_P15-30) representing 12 samples. The 0–2 cm depth sample was a solidified petroleum crust.

Soils (except ETV0_P0-2) were sieved at 4 mm, aliquots of fresh soil sample were kept at 4 °C until the return to the laboratory for mineralization activity measurements and estimation of the percentage of humidity. The rest was immediately frozen at -80 °C for further analyses (DNA and organic extractions). Texture (NF X 31–107), total nitrogen and organic carbon content (NF ISO 13,878 and NF ISO 10,694) and pH (in water, NF ISO 10,390) were measured at the LAS INRAE at Arras (France).

### Mineralization activities under aerobic and anaerobic conditions

Two replicates of each soil samples (10 g fresh weight corresponding to 5.8 to 7.8 g dry weight) collected in May 2016 on longitudinal transects were incubated into 125 ml glass flasks sealed hermetically with Teflon septum. One replicate was incubated under aerobic condition (ambient atmosphere) and the other under anaerobic condition by flushing and replacing the atmosphere by N_2_/H_2_ (95:5) gas mix. Empty control flasks without soil (similar atmosphere than for aerobic and anaerobic tests) were run in the same way to correct CO_2_ measurements. Flasks were incubated at 24 °C for 7 days. Carbon dioxide (CO_2_) release was measured after 1, 2, 3, 4 and 7 days on a 2 ml portion of the flask atmosphere (sampled using a syringe) by infrared spectrophotometer (Binos 1004, absorption at 2325.6 cm^−1^)^25^. The mineralization activities were represented by the produced CO_2_ expressed as carbon mass per gram of dry weight soil per day (µg C g^−1^ d^−1^).

### Organic extraction and hydrocarbon measurement

Solvent extractions were performed using an automated solvent extractor Dionex® ASE 350. Before extraction procedure, activated copper powder (1 g) and Na_2_SO_4_ (1 g) were added to 2 to 10 g of soil in the extraction cells to remove the molecular sulphur and the residual water, respectively. Extractions were performed twice at 130 °C and 100 bars with HPLC grade dichloromethane (DCM) using a static time of 5 min. Organic extracts were diluted with DCM to reach 20 ml. An aliquot (3 ml) was dried in a fume hood (36 h) to quantify the mass of the extractable organic matter (EOM)^26^.

The Hydrocarbon Index (HI) was measured according to the ISO 16,703:2004 procedure using a GC-FID 7890A Agilent technologies^27^. The integration limits of the chromatogram area, corresponding to the *n*-decane (C_10_H_22_) and *n*-tetracontane (C_40_H_82_), were defined using a standard mixture (ASTM D5307). For external calibration an ISO 11,046 standardized oil was used. Twelve solutions of different concentrations (from 1 to 31 mg ml^−1^) were injected into the GC-FID in order to obtain calibration curves. The HI was estimated from integration of the area under each chromatogram and corrected by the background noise (by integrating the area chromatogram obtained with a sample of pure dichloromethane that serves as a control).

Compound molecular distribution of soil EOM and crude oil (sampled directly in the middle of the oil seep) was determined through injection in a Shimadzu gas chromatograph (GC2010) equipped with a capillary column in silica glass DB-5MS (60 m × 0.25 mm i.d. × 0.1 µm film thickness) coupled with a mass spectrometer (QP2010) operating at 70 eV in fullscan mode. The inlet temperature was set at 300 °C and injections were run in split mode (split ratio of 1:5). The oven temperature program was as followed: 70 °C for 2 min, from 70 to 130 °C at 15 °C/min, then 130–315 °C at 4 °C/min followed by an isothermal stage at 315 °C for 25 min. The carrier gas was helium at 1.5 ml.min^−1^ constant flow. Compound identification was confirmed by the mass spectra obtained with the MS detector in comparison with the NIST08 and Wiley database spectra. Carbon preference index (CPI) was calculated according to Colombo et al.^28^ with the *n*-alkanes areas obtained on the GC–MS extracted ion chromatogram (m/z 57) of EOM from the different sampling points, using the formula: CPI = 2 × (*n* − C_27_ + *n* − C_29_)/(*n* − C_26_ + 2 × *n* − C_28_ + *n* − C_30_). CPI is a ratio classically used for EOM source and contribution type^28,29^, a ratio close to one is indicator of crude oil signature and higher values (> 5) indicates biogenic contribution.

### DNA extraction, bacterial and fungal abundance and quantification of known hydrocarbon-degradation genes

Genomic DNA was extracted from *ca.* 0.5 g of the soil samples using a Fast DNA spin kit for Soil (MP Biomedicals). Genomic DNA extracts were diluted to 5 ng µl^−1^ to be used as templates for further PCR and qPCR analyses.

Quantification of bacterial and fungal communities was done using real-time PCR quantification using primer sets 968F/1401R and Fung5F/FF390R for 16S and 18S rRNA gene quantification, as described previously by Cébron et al.^30^ and Thion et al. ^31^. PAH-RHD$\mathrm{\alpha }$ genes encoding alpha subunits of ring-hydroxylating dioxygenases from Gram negative (GN) Proteobacteria and Gram-positive (GP) Actinobacteria, *alk*B genes encoding alkane monooxygenases^32^ and *bss*A genes encoding benzylsuccinate synthases were quantified using PAH-RHD$\mathrm{\alpha }$ GN and GP primers, alkB-F/R primers and 7772F/8546R primers following Cébron et al.^30^, Gielnik et al.^33^ and Winderl et al.^34^, respectively. Real-time PCR assays were performed using iQ SybrGreen Supermix (Biorad) and CFX96 apparatus (BioRad). Data were expressed as gene copy number per gram of dry weight soil thanks to ten-time dilutions (from 10^8^ to 10^2^ copies/µl) of standard plasmids.

### Bacterial 16S and fungal 18S rDNA sequencing

The V3/V4 region of bacterial 16S rRNA genes (ca. 450 bp) was amplified using S-D-Bact-0341-a-S-17 and S-D-Bact-0787-b-A-20 primers following a previously described dual-index strategy^35^ using PCR primers with an Illumina adaptor, pad and index sequences^36^. PCR reactions were performed on 2 μl of diluted gDNA using Phusion high-fidelity polymerase (Thermo Scientific). PCR reactions consisted of 31 cycles with touchdown annealing temperature for 18 cycles (63–54 °C with a decrease of 0.5 °C/cycle) and 13 cycles at 54 °C. Amplification products were checked on 1% agarose gel electrophoresis and purified using the UltraClean-htp 96Well PCR Clean-Up kit (Qiagen) following the manufacturer’s instructions. After Quant-iT PicoGreen ds-DNA Assay Kit (Invitrogen) quantification, an amplicon library was prepared (equimolar pool at 10 nM), purified on QIAquick PCR purification Kit Column (Qiagen) and sent for sequencing to the Genewiz platform (South Plainfield, NJ, USA) which used an Illumina MiSeq V2 Kit for 2 × 250 bp paired-end sequencing.

The V7–V8 region of fungal 18S rRNA genes was amplified (*ca.* 390 bp) using FR1 and FF390 primers^37^ fused with a partial sequencing adapter. This first PCR was performed using Phusion high-fidelity polymerase (Thermo Scientific). The PCR reactions consisted of 25 to 30 cycles (depending on the samples) with an annealing temperature of 54 °C. Amplification products were checked by 1% agarose gel electrophoresis before being sent to Microsynth AG (Balgach, Switzerland) for further steps namely second PCR, library preparation and Illumina MiSeq 2 × 250 bp paired-end sequencing.

Illumina MiSeq paired-end reads for 16S and 18S rDNA were deposited in the SRA database under BioProjects ID: PRJNA685399 and PRJNA686256, respectively.

### Processing of bacterial 16S and fungal 18S rDNA sequencing data

Sequence data were analysed following the MiSeq SOP procedure available in March 2017 and described in Kozich et al.^35^, using Mothur v.1.36.0^38^. Only 41 out of 42 samples were treated for 16S rDNA because sequencing failed for GUN-15 third replicate. Paired-end reads were trimmed to a minimum QScore of 20 (both 16S and 18S rRNA reads) and joined using the following criteria: no ambiguous bases and 404 bp < length < 430 bp or 348 bp < length < 359 bp for 16S or 18S rDNA paired-end reads, respectively. After, alignment of unique sequence representatives to Silva V132 fasta data (released in Dec. 2017) and pre-clustering of sequences (sequences that are within 2 nt of each other), chimeras were detected and removed using Uchime and singletons (split.abund command removing sequences represented only once among all reads) were removed. Taxonomy was assigned using the Silva V132 tax data with a cut-off = 80 and using the Wang method. Sequences affiliated to archaea, chloroplasts, unknown, mitochondria and eukaryota were removed for 16S rDNA analysis, while sequences affiliated to archaea, chloroplasts, unknown, mitochondria and bacteria were removed for 18S rDNA analysis. After uncorrected pairwise distances calculation between aligned sequences, the bacterial and fungal sequences were clustered in Operational Taxonomic Units (OTUs) at 97% similarity, and consensus taxonomy for each OTU was determined using classify.otu. Finally, datasets were rarefied to the lowest number of sequences per sample (53,422 and 14,260 reads/sample for bacterial and fungal sequences, respectively).

The number of reads for each OTU was expressed as a percentage to express the relative proportion or relative abundance of each OTU within the total community. Alpha diversity was expressed by calculating Chao1 richness, Pielou’s evenness J’ and Shannon diversity H’ indices using Mothur.

### Bacterial functional trait inference

Functional traits were compared between increasing indicator taxa found in ETV0 and GUN0 samples (identified through TITAN2 analysis, see below) and the whole bacterial community inhabiting the control forest samples ETV15 and GUN15.

Inference of 19 bacterial traits (e.g. tolerance to pH, NaCl, temperature, preference to oxygen, Gram staining, width, length, shape, motility, pigment, spore production, trophic type and DNA GC %) was performed using the BactoTraits database^39^. The relative proportion of each trait attributes for all bacterial OTUs were calculated and attributed based on their taxonomic affiliation to get OTUs trait profiles. Based on the relative abundance of each OTU, the relative abundance of each trait attributes for the 19 traits was calculated and compared for the two different populations by Wilcoxon rank-sum test (p value < 0.05).

The potential functional pathways of the two bacterial populations were profiled using Tax4Fun^40^ (v 1.0.1) run on Galaxy (X. SIGENAE [http://www.sigenae.org/]). Differences in the level 1, level 2 and level 3 KEGG predicted functions of increasing indicator taxa and of control forest soil communities were compared and analyzed by Wilcoxon rank-sum test (p value < 0.05).

### Statistical analysis

Statistical analyses were performed using R studio version 3.6.0^41^. Values of soil parameters (extractable organic matter EOM, hydrocarbon index HI, total organic carbon C, nitrogen N and C/N ratio), mineralization activities, bacterial and fungal quantification (16S and 18S rRNA gene copy numbers), alpha-diversity estimators (Chao1, Shannon diversity and Pielou’s evenness indices), relative abundance of bacteria phyla and fungal divisions and the relative abundance of the bacterial functional trait attributes were compared among the 5 different zones (0, 2, 4, 10 and 15 m from the petroleum seeps) for both sites independently (GUN and ETV) and for different sampling depths at the ETV site. This was carried out through one-way analyses of variance (Anova) with p < 0.05, followed by Tukey HDS *PostHoc* tests.

Using the Vegan R package^42^, non-metric multidimensional scaling (NMDS) analyses were performed (‘*metaMDS*’ function) based on the Bray–Curtis dissimilarity matrix generated from bacterial and fungal relative abundances to estimate the dissimilarity in structure between all samples. For bacteria, OTUs having at least 0.1% relative abundance (≥ 54 reads) were kept for this analysis. The environmental gradients of hydrocarbon content were built on NMDS ordination space using the ‘Ordisurf’ function of the Vegan R package which used generalized addictive modelling (GAM) to overlay environmental variables^42,43^. Permutational multivariate analyses of variance (PERMANOVA) were performed with 999 permutations using the ‘*adonis’* function in Vegan R package to determine if bacterial and fungal community composition differed significantly between sites (next to the oil seeps vs. the others).

Threshold Indicator Taxa ANalysis (TITAN) was performed to identify environmental variable values (in our case hydrocarbon index values) maximizing taxa frequency and abundance using bootstrapping to identify reliable indicator taxa^44^. TITAN 2.4 package in R^45^ was used. Association was measured by taxon abundances weighted by their occurrence in each sample as for Indicator taxa determination^46^ and standardized as z-scores to facilitate cross-taxon comparison via permutation of samples along the predictor. The number of bootstraps and permutations performed in our TITAN2 analysis was 500 and 250, respectively. Analysis was performed at the OTU taxonomic level. Only OTUs observed more than 3 times across all sites were used and thus 3,306 and 861 OTUs were considered for bacterial and fungal analyses respectively. TITAN2 distinguishes declining and increasing Indicator Taxa along environmental gradient.

### Originality-significance statement

The significance and novelty of our work is the study of bacterial and fungal diversity and the identification of indicator taxa developing next to petroleum seeps and most likely involved in the hydrocarbon degradation. The originality is that these indicator taxa are well-known aerobic and anaerobic bacteria having specific functional traits making them well-adapted to hydrocarbon-contaminated environment as well as fluctuating redox conditions.

## Results and discussion

### Soil characteristics, and hydrocarbon contamination levels

Soil properties were characterized for the two sampling sites and both campaigns (Table 1). At the ETV site, nitrogen content was higher next to the oil seep and its content decreased with depth, while staying constant all over the transect at GUN site. Due to high concentration of petroleum carbon, the C/N ratio was high next to the oil seeps (c.a. 40) compared to values found at a 15 m distance (c.a.15). Finally, the pH values were also higher next to the oil seeps and decreased with distance. It was previously shown that oil contaminated soils hrabored alkalin pH^47,48^. At the first zone next to the petroleum seep, at both sites, the average quantity of extractable organic matter (119.2 and 118.6 mg EOM g^−1^ in the GUN and ETV sites, respectively), and the hydrocarbon index (HI, 73.7 and 35.3 mg g^−1^ in the GUN and ETV sites, respectively) values were significantly higher than in the samples farthest from the seeps at a distance of 10 or 15 m which revealed high oil contamination of the soil at the vicinity of the seeps. The EOM and HI values did not decrease gradually with distance but dropped by more than 90% from the second sampling zone (a 2 m distance from the oil seeps). In opposite way, an increase in the carbon preference index (CPI) with increasing distance from the oil seep (Table 1), was shown. CPI is a ratio classically used for EOM source and contribution type^28,29^. The low ratio (around 1) as measured next to the oil seeps is indicator of crude oil signature and higher values (> 5) indicates a major contribution of biogenic compounds to the soil organic matter. For both sites, samples closest to the oil seeps were dominated by *n*-alkanes similar to those of the crude oil (Figure S1); this strong crude oil contribution in the EOM fingerprint decreased as the sampling points get further from the oil seepage. The EOM molecular distribution became dominated by *n*-alkanols and fatty aldehydes with even number of carbon atoms, and *n*-alkanes with odd number of carbon atoms (Figure S1), characteristic of biogenic samples and higher plant contribution^28,29^ commonly found in natural soils. At the farthest sampling points from the oil seeps (ETV15 and GUN15) petroleum signature completely disappeared (Figure S1). Samples from the second sampling campaign showed a similar gradient of hydrocarbon contamination with depth but at a smaller scale, highlighting higher contamination in the surface crust (108.9 and 42.3 mg g^−1^ for EOM and HI, respectively) compared to the samples taken at a depth of 15 and 30 cm (Table 1). Same gradients were observed for the total organic carbon content with distance and depth (Table 1).

**Table 1:** Pollution and soil characteristics along longitudinal and depth gradients.

	Sampling date	Distance from oil seep (m)	Depth (cm)	Extractable organic matter, EOM (mg g^−1^)	Hydrocarbon index, HI (mg g^−1^)	Carbon preference index, CPI	TOC (mg g^−1^)	N (mg g^−1^)	C/N	pH
GUN0	May 2016	0	0–5	119.2 ± 21.9 ^a^	73.7 ± 26.3 ^a^	1.0	142	3.5	40.3	6.88
GUN2		2	0–5	11.7 ± 0.9 ^b^	3.2 ± 0.2 ^b^	6.2	80	4.8	16.6	7.00
GUN4		4	0–5	3.0 ± 0.2 ^b^	0.9 ± 0.1 ^b^	10.0	61	4.1	14.8	6.05
GUN10		10	0–5	3.2 ± 0.8 ^b^	1.0 ± 0.3 ^b^	10.8	42	2.8	15.1	5.76
GUN15		15	0–5	1.8 ± 0.2 ^b^	0.4 ± 0.1 ^b^	9.7	43	3.0	14.4	5.51
ETV0	May 2016	0	0–5	118.6 ± 44.6 ^a^	35.3 ± 13.3 ^a^	1.0	154	3.5	43.8	6.09
ETV2		2	0–5	48.7 ± 20.2 ^ab^	11.2 ± 5.6 ^ab^	2.3	103	3.8	27.0	6.14
ETV4		4	0–5	20.9 ± 14.4 ^ab^	3.7 ± 2.5 ^b^	3.1	57	2.4	23.7	6.03
ETV10		10	0–5	6.3 ± 4.4 ^b^	0.5 ± 0.2 ^b^	6.4	23	1.4	16.9	5.77
ETV15		15	0–5	14.8 ± 9.4 ^ab^	0.5 ± 0.1 ^b^	6.2	27	1.7	15.7	5.19
ETV0_P0-2	May 2017	0	0–2	108.9 ± 9.0 ^ab^	42.3 ± 1.9 ^a^	na	141 ± 2 ^a^	3.1 ± 0.2 ^a^	46.2 ± 2.8 ^ab^	na
ETV0_P2-5		0	2–5	113.0 ± 18.2 ^a^	24.9 ± 3.1 ^b^	na	142 ± 19 ^a^	3.8 ± 0.5 ^a^	37.6 ± 1.1 ^ab^	na
ETV0_P5-15		0	5–15	73.0 ± 27.3 ^bc^	11.6 ± 5.1 ^c^	na	42 ± 9 ^b^	1.7 ± 0.4 ^ab^	25.4 ± 0.8 ^b^	na
ETV0_P15-30		0	15–30	31.7 ± 19.5 ^c^	5.6 ± 3.7 ^c^	na	13 ± 3 ^b^	0.4 ± 0.2 ^b^	51.5 ± 19.6 ^a^	na

Mean (n = 3) and standard error (when no standard error is indicated it means that only one value was available, i.e. measurement was performed on a mean sample constituted by a pool of the 3 replicates). Letters indicate significant differences (p < 0.05) among sampling zones of the same site and sampling date (one-way anova followed by Tukey HSD PostHoc test). GUN: Gunstett site, ETV: Etang-vert site. TOC: Total organic carbon, N: total nitrogen, C/N: ratio of carbon to nitrogen.

### Microbial activity and abundance

To evaluate the functional potential of hydrocarbon degradation, we measured the microbial activity of organic matter mineralization under aerobic and anaerobic conditions. Both aerobic and anaerobic microbial mineralization activities were detected. Mineralization activity was 3–7 times greater under aerobic than under anaerobic conditions for both sites (Table 2). Both aerobic and anaerobic mineralization activities were significantly higher next to the oil seeps at the first sampling zone compared to the rest of the transect, and decreased with depth at ETV site. Significant positive Pearson correlations (p < 10^–5^) were found between organic matter mineralization activities and HI values (r = 0.61 and 0.52 for aerobic and anaerobic mineralization activities, respectively). As much of the organic matter found next to the oil seep is composed of petroleum hydrocarbons we can assume that microbial activities were mainly dedicated to hydrocarbon degradation both in aerobic and anaerobic conditions.

**Table 2:** Microbial mineralization activities, abundances (16S and 18S rRNA gene copy numbers for bacteria and fungi, respectively) and alpha-diversity indices (Chao1 richness, Shannon diversity and Pielou’s evenness) along longitudinal and depth gradients.

	Sampling date	Distance from oil seep (m)	Aerobic mineralization CO_2_ production (µg C-CO_2_ g^−1^ d^−1^)	Anaerobic mineralization CO_2_ production (µg C-CO_2_ g^−1^ d^−1^)	Bacteria				Fungi			
					16S rRNA gene copies (× 10^10^ g^−1^)	Chao 1 richness	Shannon diversity H’	Pielou’s evenness	18S rRNA gene copies (× 10^8^ g^−1^)	Chao 1 richness	Shannon diversity H’	Pielou’s evenness
GUN0	May 2016	0	248 ± 90 ^a^	61 ± 5 ^a^	8.7 ± 3.3 ^ab^	6532 ± 441	6.0 ± 0.4 ^b^	0.70 ± 0.04 ^b^	10.0 ± 1.6	349 ± 10	3.05 ± 0.16 ^b^	0.55 ± 0.03 ^b^
GUN2		2	94 ± 7 ^b^	31 ± 1 ^b^	9.6 ± 2.2 ^ab^	7292 ± 676	7.3 ± 0.2 ^a^	0.83 ± 0.02 ^a^	12.8 ± 4.6	429 ± 56	3.72 ± 0.22 ^a^	0.64 ± 0.02 ^a^
GUN4		4	76 ± 13 ^b^	26 ± 5 ^b^	10.0 ± 2.1 ^a^	7988 ± 435	7.2 ± 0.4 ^a^	0.82 ± 0.04 ^a^	26.7 ± 16.1	448 ± 79	3.29 ± 0.39 ^ab^	0.57 ± 0.05 ^ab^
GUN10		10	61 ± 25 ^b^	26 ± 9 ^b^	5.1 ± 0.3 ^b^	7156 ± 573	7.2 ± 0.2 ^a^	0.82 ± 0.02 ^a^	14.7 ± 3.9	426 ± 6	2.55 ± 0.18 ^c^	0.45 ± 0.03 ^c^
GUN15		15	47 ± 11 ^b^	18 ± 4 ^b^	6.0 ± 1.6 ^ab^	7697 ± 158	7.3 ± 0.0 ^a^	0.84 ± 0.01 ^a^	14.4 ± 6.7	460 ± 65	3.45 ± 0.14 ^ab^	0.59 ± 0.02 ^ab^
ETV0	May 2016	0	192 ± 39 ^a^	32 ± 12 ^a^	6.9 ± 1.7	4683 ± 2150 ^b^	5.1 ± 1.3 ^b^	0.62 ± 0.13 ^b^	8.2 ± 3.8	450 ± 44	3.28 ± 0.38	0.56 ± 0.06
ETV2		2	134 ± 72 ^ab^	38 ± 7 ^a^	8.4 ± 1.7	8630 ± 711 ^a^	7.5 ± 0.1 ^a^	0.84 ± 0.02 ^a^	48.1 ± 61.3	421 ± 154	2.79 ± 1.87	0.47 ± 0.30
ETV4		4	56 ± 46 ^b^	18 ± 8 ^ab^	8.5 ± 3.0	7908 ± 944 ^a^	7.3 ± 0.3 ^a^	0.83 ± 0.03 ^a^	12.2 ± 0.1	498 ± 69	3.59 ± 0.55	0.60 ± 0.08
ETV10		10	44 ± 9 ^b^	13 ± 7 ^b^	5.6 ± 1.0	8670 ± 882 ^a^	7.5 ± 0.3 ^a^	0.84 ± 0.02 ^a^	7.9 ± 2.3	562 ± 57	3.78 ± 0.06	0.62 ± 0.00
ETV15		15	42 ± 7 ^b^	7 ± 5 ^b^	6.1 ± 0.7	7198 ± 1361 ^a^	7.1 ± 0.2 ^a^	0.81 ± 0.01 ^a^	11.4 ± 1.4	492 ± 56	3.50 ± 0.21	0.59 ± 0.02
ETV0_P0-2	May 2017	0	207 ± 29 ^a^	31 ± 3 ^a^	10.6 ± 2.0 ^a^	3539 ± 732 ^b^	4.2 ± 0.6 ^c^	0.53 ± 0.06 ^c^	6.5 ± 1.9	355 ± 5	3.03 ± 0.23	0.54 ± 0.05
ETV0_P2-5		0	88 ± 16 ^b^	24 ± 6 ^ab^	6.1 ± 0.9 ^ab^	4988 ± 479 ^a^	5.1 ± 0.3 ^b^	0.61 ± 0.03 ^b^	3.0 ± 0.5	395 ± 35	2.88 ± 0.62	0.50 ± 0.10
ETV0_P5-15		0	51 ± 35 ^bc^	16 ± 8 ^b^	5.1 ± 2.2 ^b^	5204 ± 921 ^a^	6.4 ± 0.4 ^a^	0.76 ± 0.04 ^a^	5.2 ± 1.6	303 ± 56	2.15 ± 0.70	0.40 ± 0.11
ETV0_P15-30		0	15 ± 5 ^c^	4 ± 1 ^c^	2.3 ± 0.6 ^c^	5823 ± 160 ^a^	6.8 ± 0.0 ^a^	0.80 ± 0.00 ^a^	1.9 ± 0.3	347 ± 74	2.46 ± 0.44	0.44 ± 0.06

Samples from May 2016 were sampled in surface (0–5 cm depth) and samples from May 2017 were sampled at 4 depth (0–2, 2–5, 5–15 and 15–30 cm depth). Mean (n = 3) and standard deviation. Letters indicate significant differences (p < 0.05) among sampling zones of the same site and sampling date (one-way anova followed by a Tukey HSD PostHoc test). GUN: Gunstett site, ETV: Etang-vert site. Mineralization activities and rRNA gene copies are expressed as gram of dry weight soil.

The distance to the petroleum seep was not found to significantly influence the number of bacteria and fungi (Table 2). We quantified 5 × 10^10^ to 10 × 10^10^ copies of 16S rRNA genes per gram of soil in both soil transects. Fungal 18S rRNA gene copy number was 100 times lower than for 16S rRNA one. The quantification of bacteria showed a significant decrease with soil depth from 10 × 10^10^ to 2 × 10^10^ 16S rRNA gene copy numbers per gram of soil from the surface (0–2 cm) to the 15–30 cm depth samples.

### Quantification of known hydrocarbon-degradation genes

The well-known bacterial genes encoding PAH dioxygenase (PAH-RHD$\mathrm{\alpha }$), alkane monooxygenase (*alk*B) and benzylsuccinate synthase-like (*bss*A) were quantified to estimate the functional population potentially involved in hydrocarbon degradation in aerobic and anaerobic conditions (Table S1). No significant difference in PAH-RHD$\mathrm{\alpha }$ and *alk*B gene abundance was shown across the transects even if these genes involved in aerobic hydrocarbon degradation tended to be in higher quantity next to the oil seep in samples from ETV site. At this site, the abundance of PAH-RHD$\mathrm{\alpha }$ genes from both Gram-negative Proteobacteria and Gram-positive Actinobacteria was significantly higher in surface crust (0–2 cm) than in the deeper samples (5–30 cm). However, when expressed as percentage compared to 16S rRNA genes, PAH-RHD $\mathrm{\alpha }$ genes represent less 0.02% while *alk*B genes could represent up to 1.0% next to the oil seep. Even if well-known Proteobacteria and Actinobacteria possess these functional genes^10,30,32^, it’s more likely that most taxa involved in hydrocarbon degradation in situ would possess other suites of genes because they were detected in really low quantity. It was previously showed that for active taxa belonging to PAH-degrading consortia identified through stable isotope probing, the ratio of PAH-RHD$\mathrm{\alpha }$ relative to 16S rRNA genes only accounted for 1.2 to 5.9%^49^. Similarly, genes involved in all steps of aromatic compounds metabolism represented less than 5% of the metagenome of an active PAH-degrading consortia where most of the taxa were not involved in the first hydroxylating step but in intermediate metabolite degradation^50^. Targeting PAH-RHD$\mathrm{\alpha }$ and *alk*B genes involved in this first step is not sufficient to evaluate the abundance of bacteria involved in all steps of hydrocarbon mineralization. When targeting *bss*A genes involved in anaerobic hydrocarbon degradation, a significant higher abundance of these genes was detected in DNA extracted from sites close to the oil seeps in both ETV and GUN sites and in the surface petroleum crust in ETV site (Table S2). However, these genes also represent a low percentage compared to 16S rRNA genes (less than 0.004%). Even if the identification of individual metabolic genes involved in oil degradation in situ sometime showed correlation between functional-population abundance and contamination level^30,34^, here it was not sufficient to conclude about functional pathways involved in oil degradation. Metagenomic analysis could be helpful in resolving this question but unknown genes and pathways (not annotated) as well as the metabolic complementarity of the taxa could prevent the analysis of such complex metagenomes, especially as there seem to be both anaerobic and aerobic processes taking place in our study sites.

### Bacterial and fungal community : alpha-diversity indices and composition

To help us assess the impact of hydrocarbons on the microbial community structure and composition, 16S rDNA and 18S rDNA sequencing were performed. After quality filtering, a total of 5,187,772 and 2,485,385 paired-end sequences were retrieved from the 16S rDNA and 18S rDNA libraries, ranging from 53,422 to 122,937 reads per sample for 16S rDNA and from 4,016 to 79,581 reads per sample for 18S rDNA. After sub-sampling to 53,422 and 14,260 reads per samples for 16S and 18S rDNA libraries, respectively, both 16S and 18S rDNA data were analyzed for 41 samples out of 42 (GUN-15 third replicate failed for 16S rDNA sequencing and ETV-15 s replicate was removed for 18S rDNA analysis because of a too low number of sequences). No general trend was found for fungal alpha-diversity indicators (Table 2), either according to the distance to the oil seep or in depth. However, on both sites, bacterial richness (Chao1), Shannon diversity and Evenness indices were lower next to the oil seeps compared to all other sampling distance and also in the surface crust compared to the other depth samples (Table 2). Negative Pearson correlations were observed between EOM, HI and TOC values and the 3 alpha-diversity indices (Chao1, Shannon and Evenness) describing bacterial community composition (Table 3) for the 2 sites. Similarly, a lower bacterial taxonomic richness was observed in oil plume from Deepwater horizon spill^6^. This observation indicate that mostly hydrocarbon-degrading bacteria were enriched next to the oil seeps and that bacteria sensitive to petroleum contamination disappeared as shown previously following oil contamination due to pipeline rupture^51^.

**Table 3:** Pearson correlation coefficients between the 3 bacterial alpha-diversity indicators and the extractable organic matter, hydrocarbon index and total organic carbon values for longitudinal and depth gradients.

		Chao1	Shannon H’	Evenness
GUN (longitudinal)	Extractable organic matter	−0.65*	−0.91***	−0.91***
	Hydrocarbon index	−0.59*	−0.85***	−0.85***
	TOC	−0.57*	−0.83***	−0.83***
ETV (longitudinal)	Extractable organic matter	−0.68**	−0.85***	−0.88***
	Hydrocarbon index	−0.71**	−0 87***	−0.89***
	TOC	−0.55*	−0.67**	−0.69**
ETV (depth)	Extractable organic matter	−0.72**	−0.74**	−0.72**
	Hydrocarbon index	−0.86***	−0.91***	−0.90***
	TOC	−0.70*	−0.93***	−0.93***

P values are given by * for < 0.05, ** for p < 0.01 and *** for p < 0.001.

**Figure 2:**
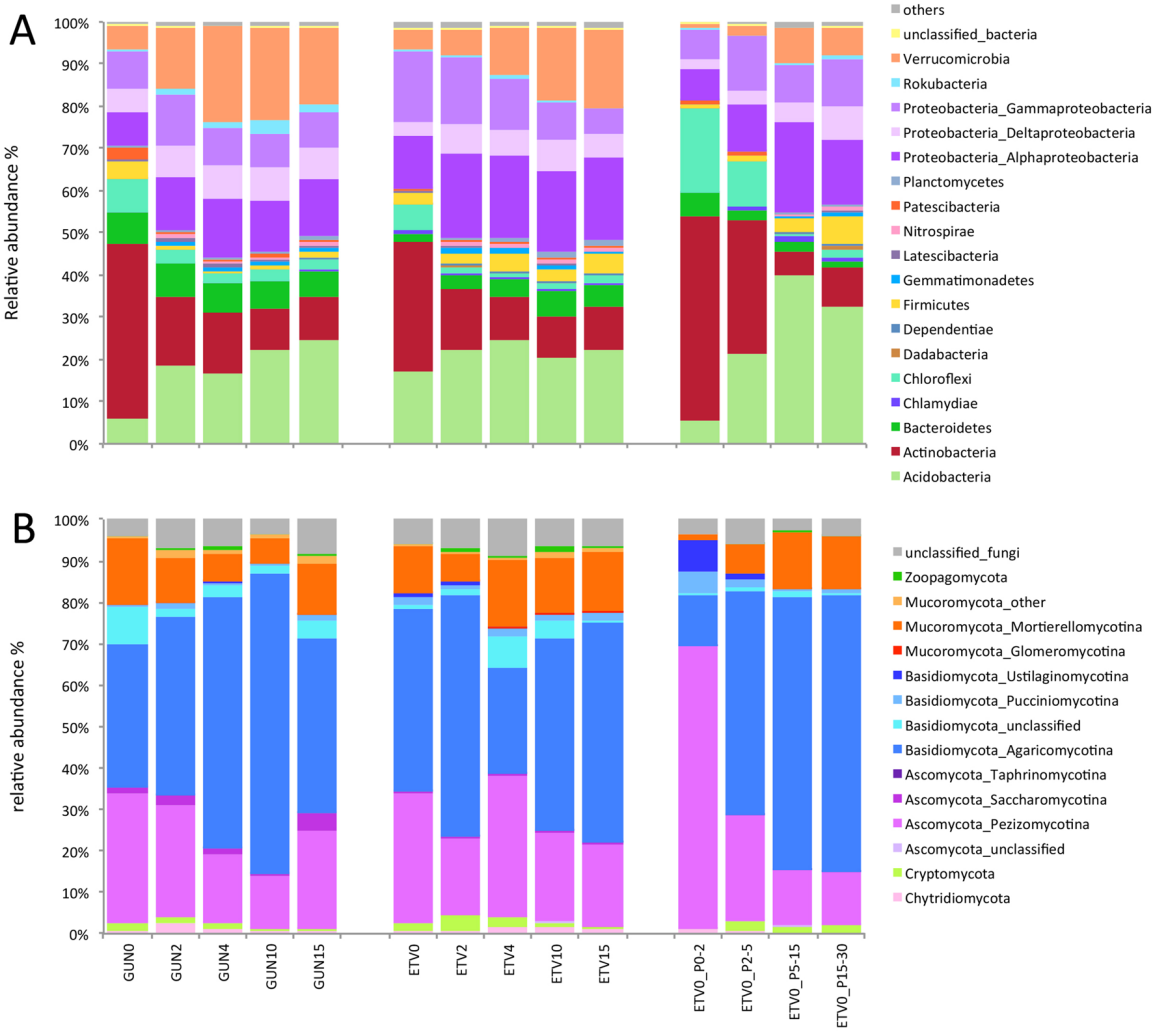
Changes in relative abundance of bacterial phyla (**A**) and fungal sub-divisions (**B**) in GUN and ETV sites according to the distance from the petroleum seeps (0, 2, 4, 10 and 15 m) and depth (P0-2, P0-5, P2-5, P5-15 and P15-30 cm) at ETV site for the 0 m distance. Data are means (n = 3). Different classes of Proteobacteria (plot A) and sub-divisions of Mucoromycota, Basidiomycota and Ascomycota (plot B) were shown. The group “Others” (plot A) includes phyla below 0.5% (267 reads) of relative abundance and affiliated to Armatimonadetes, BCR1, Caldiserica, Calditrichaeota, Cyanobacteria, Deferribacteres, Deinococcus-Thermus, Elusimicrobia, Entotheonellaeota, FBP, FCPU426, Fibrobacteres, Firestonebacteria, Fusobacteria, GAL15, Halanaerobiaeota, Hydrogenedentes, Kiritimatiellaeota, Lentisphaerae, Margulisbacteria, Nitrospinae, Omnitrophicaeota, Schekmanbacteria, Spirochaetes, TA06, Tenericutes, unclassified_bacteria, WPS_2, WS2, WS4 and Zixibacteria.

**Figure 3:**
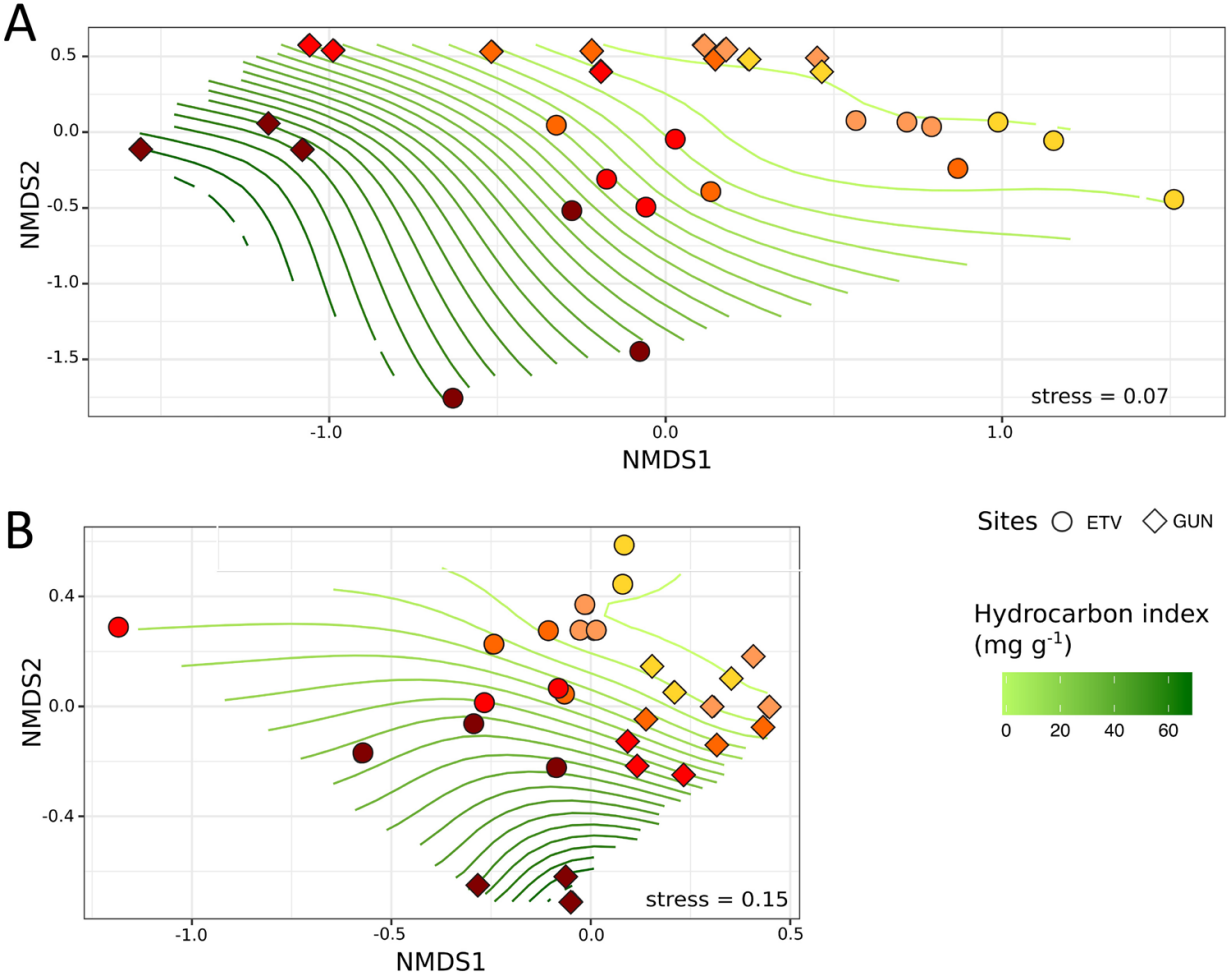
Ordisurf model showing the distribution of hydrocarbon index (HI) level over the NMDS ordination of bacterial (**A**) and fungal (**B**) community structures at the OTU level. The hydrocarbon gradient is visualized through green curves. ETV and GUN sites are differentiated through circles and diamonds, respectively. Different sampling zones are visualized thanks to colour gradients from dark red (0 m, close to the oil seep), red (2 m), orange (4 m), light orange (10 m) and yellow (15 m).

Overall no significant bacterial community composition difference (Wilcoxson test, p = 0.543) was detected between GUN and ETV sites at the phylum level. The distance to the petroleum seep positively or negatively influenced many bacterial phyla (Fig. 2A). The relative abundance of Actinobacteria, Chloroflexi, Firmicutes and Kiritimatiellaoeota members was significantly higher next to the oil seep and decreased with distance on GUN site (p ≤ 0.003). For the two first phyla the same tendency was observed on ETV site along the longitudinal gradient (p ≤ 0.016) and with depth with a significantly higher proportion (p < 0.0001) of these phyla in the surface petroleum crust than at 5–30 cm depth. It was previously shown that Actinobacteria and Firmicutes dominated the bacterial community in soil after oil pipeline rupture^52^ and that Chloroflexi and Firmicutes relative abundance correlated with diesel-contamination level^53^. *Chloroflexi* can degrade petroleum hydrocarbons by anaerobic respiration. The relative abundance of Verrucomicrobia, Alpha-Proteobacteria, Planctomycetes, Elusimicrobia and Fibrobacteres (the two latter classified in the group “others”) members (Fig. 2A) increased significantly when moving away from the oil seep for both sites (p ≤ 0.047). Although no trend was found in the literature at the phylum level, some members of these phyla are most likely sensitive to oil contamination. For both GUN and ETV sites, the bacterial community structures at the OTU level were found to be changed according to the petroleum contamination of soil samples as confirmed using NMDS analysis where the hydrocarbon concentration gradient was overlapped (Fig. 3A). On the first component, samples were separated according to the hydrocarbon content and the two sites were separated on the 2nd component. At OTU level, the bacterial community composition was significantly different among the different sampling areas (Permanova, p = 0.001). Previous studies have shown such structuration of the bacterial communities according to hydrocarbon contamination level^54,55^.

Overall no significant fungal community composition difference (Wilcoxson test, p = 0. 861) was detected between GUN and ETV sites at the division level. Fewer changes were observed for the fungal community composition at the division and sub-division levels along longitudinal and depth gradients (Fig. 2B). Although the relative abundance of members of the Taphrinomycotina sub-division (Ascomycota) was very low (< 0.3%), a significant increase was observed next to the oil seep (p = 0.014) and in the petroleum surface crust (p = 0.042). Similarly, the relative abundance of Ustilaginomycotina, Pucciniomycotina (both Basidiomycota) and Pezizomycotina (Ascomycota) members was significantly higher in the surface petroleum crust and decreased with depth (p ≤ 0.032). Pezizomycotina was found to be dominant^56^ and were isolated^57^ in diesel-contaminated soil, maybe because of their hydrocarbon-degradation pathways^58^. Modification of the fungal community structure at the OUT level was less pronounced than for bacteria, but a similar gradient was observed according to the hydrocarbon content (Fig. 3B). The lower impact of on fungal community can suggest that hydrocarbons are less toxic to fungi, that fungi can cope with hydrocarbon contamination or that they can benefit from hydrocarbon compounds for their growth being well adapted to the degradation of complex carbon substrates. Fungi with their multiple enzymatic activities (hydrolytic enzymes or oxidases such as peroxidases or phenol oxidases) are able to degrade organic pollutants such as aliphatic hydrocarbons and PAHs^59^.

### Bacterial and fungal indicator taxa

**Figure 4:**
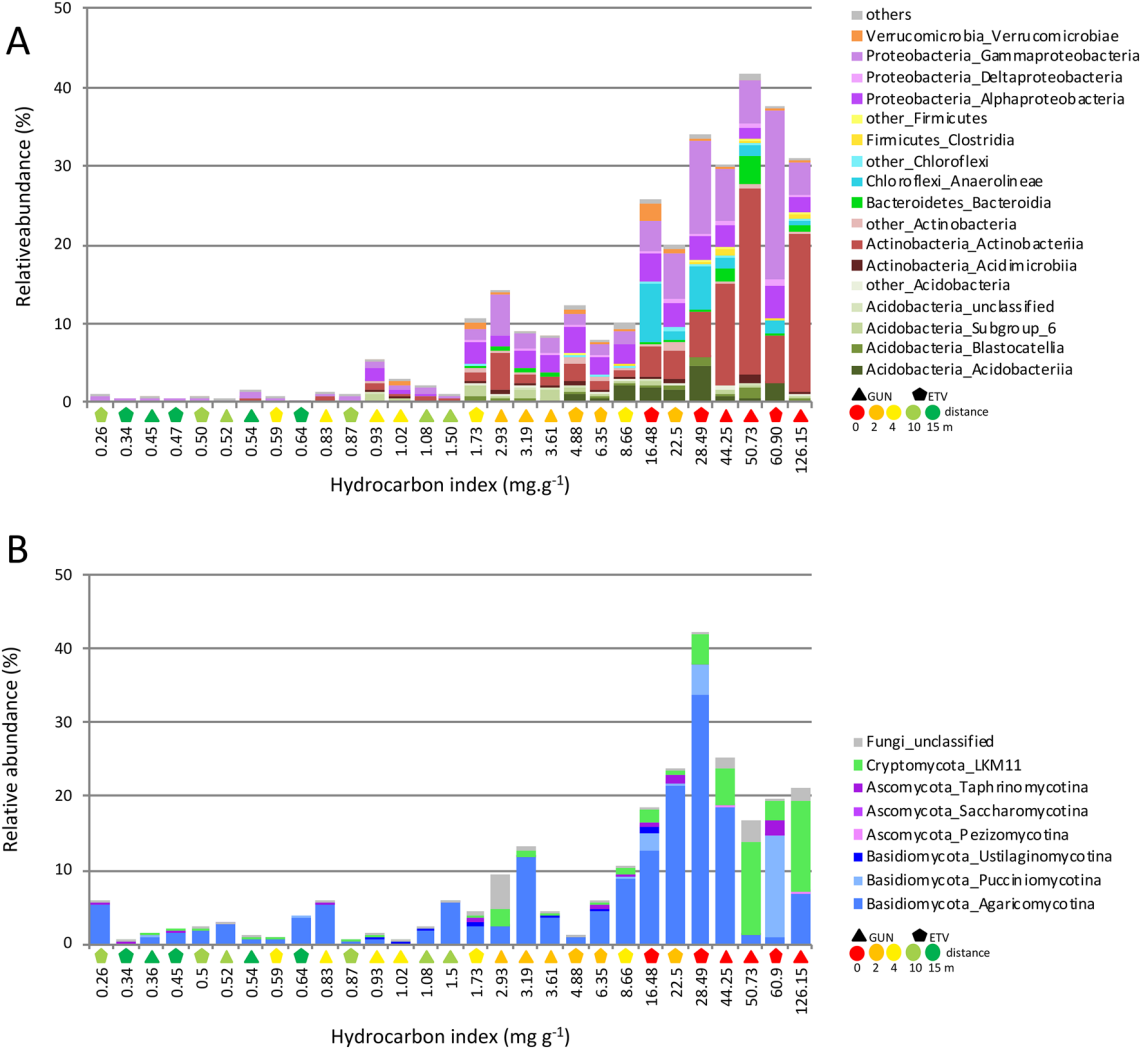
Relative abundance of increasing bacterial (**A**) and fungal (**B**) indicator taxa (summed at the class and sub-division levels for bacteria and fungi, respectively) determined using TITAN2 along the gradient of oil contamination for the combination of both ETV and GUN sites. ETV and GUN sites are symbolised by pentagon and triangle symbols, respectively, and distance to the oil seep is shown with colour gradient (from red to green). At the OTU level, 281 and 61 bacterial and fungal indicator taxa were sorted with purity and reliability parameters both > 0.95. For the complete list of bacterial and fungal OTU found as indicator taxa see Table S2 and S3, respectively. The relative abundances of OTU belonging to similar classes were summed. The group “Others” comprise classes having less than 3 representative OTUs and were affiliated to Chlamydiae, Melainanabacteria (Cyanobacteria phylum), Dadabacteriia, Babeliae (Dependentiae phylum), Gemmatimonadetes, Latescibacteria, Nitrospira, Saccharimonadia (Patescibacteria phylum) and Rokubacteria NC10 classe. The group “other_Acidobacteria” comprise OTU affiliated to Holophagae, subgroup_5 and subgroup_22 classes. The group “other_Actinobacteria” comprise OTU affiliated to Thermoleophilia and Coriobacteriia classes. The group “other_Chloroflexi” comprise OTU affiliated to Chloroflexia, TK10 and JG30-KF-CM66 classes. The group “other_Firmicutes” comprise OTU affiliated to Bacilli and BRH-c20a classes.

As bacterial and fungal community composition are shaped by petroleum contamination, indicator taxa (IT) relative to the hydrocarbon level were sought to further understand which microorganisms are tolerant/sensitive to oil contamination, potentially involved in hydrocarbon degradation or benefiting of degradation metabolites. Data from both sampling sites were combined and indicator taxa were sorted using TITAN2, a well-adapted tool for gradient studies. Bacterial and fungal indicator taxa increasing (Fig. 4) or decreasing (Figure S2) along the hydrocarbon contamination gradient (HI values) were identified. As both bacterial and fungal indicator taxa were identified, we confirmed our hypothesis that different groups of bacteria and fungi with complementary metabolic properties may coexist and potentially cooperate at the vicinity of the oil seeps for oil degradation^60^. The presence of all these microorganisms with varied metabolisms should form an efficient barrier against dissemination of the petroleum contamination under fluctuating environmental conditions.

TITAN2 sorted 281 and 664 bacterial OTUs as indicator taxa which were found to increase and decrease respectively with HI values (the complete list is available in Table S2). The relative abundance of these indicator taxa reached 30–40% when getting closer to or further from the oil seeps for increasing and decreasing taxa respectively. Among the bacterial IT increasing next to the oil seeps, we found 110 OTUs belonging to Proteobacteria, 51 to Acidobacteria, 39 to Actinobacteria, 16 to Chloroflexi, 16 to Firmicutes and 14 to Bacteroidetes (Fig. 4A). Various IT belonging to lineages of anaerobic or facultative anaerobic bacteria, had their relative abundance increasing next to the oil seeps. Members of the Acidithiobacillaceae family were identified. In this family, bacterial strains belonging to the *Acidithiobacillus* genera are chemolithoautrophes that can oxidize elemental sulphur or oxidize minerals containing ferrous iron^61^. Among the Rhodocyclaceae family, OTUs belonging to *Dechloromonas* and *Sulfuritalea* genera were identified as increasing IT. These genera were previously detected in hydrocarbon-polluted sites^62^ and could degrade a wide variety of aromatic compounds under anaerobic nitrate-reducing conditions^63–65^ through potentially original pathways^66^. Moreover, *Sulfuritalea* are also well recognized as chemolithoautotrophic sulfur-oxidizing bacteria^67^. Some OTUs affiliated to the Chloroflexi and belonging to the *Anaerolineae* were also identified as increasing IT. This lineage is composed of bacteria known as strictly anaerobic fermentative and sulphate-reducing bacteria, previously found in hydrocarbon-rich sludge samples^68^ and in anaerobic alkane-degrading culture^69^. Other IT mostly known as aerobic or facultative aerobic taxa had their relative abundance increasing next to the oil seeps. OTUs belonging to Gamma-Proteobacteria were identified, they are affiliated to the Porticoccaceae family and to *Immundisolibacter*, *Rhodanobacter* and *Pseudoxanthomonas* genera. Members of Porticoccaceae family have been described as aromatic-hydrocarbon degraders^70^ and alkane-degraders^71^, but they also possess in their genomes a high number of secondary alkane-degrading genes^72^. *Immundisolibacter* strain have been isolated from aerobic bioreactor for its capacity to degrade high-molecular-weight PAHs^73^. Even if *Rhodanobacter* was previously identified as a member of a PAH-degrading consortium^74^ it would more likely degrade intermediate metabolites^75^ because no PAHs degradation gene have been detected in sequenced genomes^76^. The *Pseudoxanthomonas* species have been found to degrade different PAHs^77,78^ and BTEX compounds^79^ and some strains could produce a rhamolipid surfactant that enhances the hydrocarbon degradation^80^. Two types of Alpha-Proteobacteria were also identified as aerobe increasing IT. The Methyloligellaceae members that are well-known methylotrophs^81^, and the *Parvibaculum* genus possessing genes involved in alkane utilisation^82^ that was detected *in situ*^83^ and in aerobic enrichment culture degrading crude oil^84^. Well known aerobic alkanes and PAHs degraders, such as the Actinobacteria belonging to *Williamsia*^85,86^, *Cellulomonas*^87,88^, *Agromyces*^89^ and *Mycobacterium*^90^ genera, were almost undetected in the forest soil but became abundant next to the oil seeps and identified as increasing IT. These Actinobacteria are equipped with hydroxylase enzymes such as monooxygenases and dioxygenases, to initiate the aerobic oxidation of hydrocarbon molecules^10,91^. In addition, some of these genera can also produce exopolysaccharides (EPS) which serve as bioemulsifiers such as glycolipides or curdlan-like EPS produced by *Cellulomonas spp.*^88,92^. Finally, two types of Acidobacteria appeared as increasing IT, the members of Solibacterales order with undemonstrated hydrocarbon-degradation capacities but that were previously found in oil contaminated soil and sediments^93,94^ and the members of the Acidobacteriales order that were identified as aerobic benzene-degraders using stable isotope probing technique^95^.

As shown before, the fungal community structure was less impacted by petroleum contamination and thus a low number of OTUs indicator taxa was found. TITAN2 sorted 61 and 40 fungal OTUs as indicator taxa which were found to increase (Fig. 4B) and decrease (Figure S2) respectively with hydrocarbon index values (complete list is available in Table S3). The relative abundance of the indicator taxa which increased with the gradient (Fig. 4B) was highly variable from one sample to another (1 to 42%). However, two fungal OTUs have higher relative abundance close to the oil seep, one belonging to *Lactarius* genus (Basidiomycota, *Agaricomycetina*) and another belonging to Cryptomycota LKM11. *Lactarius* genera are well-known forest soil symbiotroph ectomycorrhizal fungi but can also be facultative saprotroph^96^. *Lactarius* species increased up to 33.8% close to the oil seep indicating an essential role in the biodegradation of hydrocarbons. Indeed, the role of *Agaricomycetes* in bioremediation of crude oil-contaminated soil was demonstrated^97^ and the in vitro degradation of various hydrocarbons was reported for many Basidiomycetes^98^. Cryptomycota LKM11 represented 1.8 to 12.7% at the sampling sites close to the oil seeps. This fungal sub-division was recently found in PAH contaminated soils^99^, anoxic and hydrocarbon-enriched oil sand tailings ponds^100^ or creosote polluted site^101^.

### Functional traits of the increasing bacterial indicator taxa

**Figure 5:**
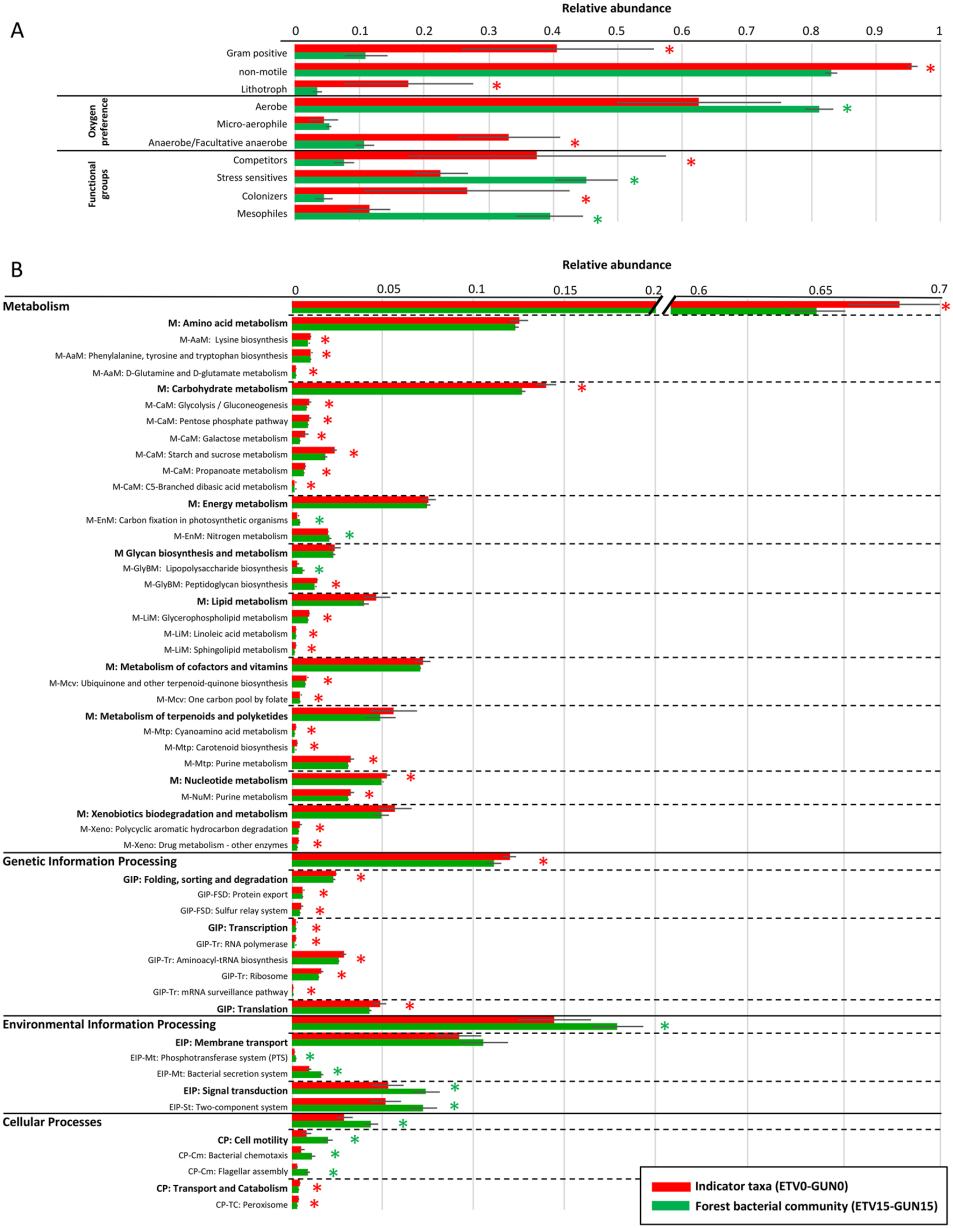
Functional traits of the increasing bacterial indicator taxa (detected in ETV0 and GUN0, in red) compared to control forest soil bacteria (detected in ETV15 and GUN15, in green) based on Bactotraits (A) and Tax4Fun (B) analyses. Only traits with significant differences between the 2 populations were represented (except for few functional pathways at the Level2 on plot B) and labelled with a * meaning that Wilcoxon rank sum tests had a p value < 0.05. Trait attributes are expressed as mean percentage (n = 6 for increasing Indicator taxa, and n = 5 for forest bacterial community) and ± standard deviation. (**A**) Morphological (Gram staining: the rest are Gram negatives, and motility: the rest are motile bacteria possessing flagella) and physiological (trophic type as lithotroph, i.e. bacteria that obtains its energy (electron donor) from inorganic compounds (iron, sulfur…) in opposition to organotroph, and oxygen preference: all attribute classes are shown) and affiliation to functional groups (5 functional groups were defined, no difference was found for stress-tolerant group) were assessed using BactoTraits (Cébron et al. 2021). (**B**) Genomic traits, i.e. KEGG functional pathways shown at Level 1, Level 2 and Level 3. Only functions representing more than 0.1% were represented. *M: Metabolism, GIP: Genetic information processing, EIP: Environmental Information Processing and CP: Cellular Processes*.

Functional traits were predicted from BactoTraits^39^ (morphological and physiological traits and affiliation to functional groups) and Tax4Fun^40^ (KEGG functional pathways) tools to better characterize the bacterial indicator taxa increasing and dominant next to the oil seeps (Fig. 5). We hypothesized that increasing bacterial IT possess specific functional traits making them tolerant to hydrocarbon contamination and giving them a functional advantage to survive and grow next the oil seeps.

Among the bacterial IT increasing next to the oil seeps, a higher proportion of Gram positive and non-motile bacteria were found (Fig. 5A) and confirmed by predicted functions showing that functional pathways related to cell motility (bacterial chemotaxis and flagellar assembly) were detected in lower proportion (Fig. 5B). Bacteria characterized as “competitors” and “colonizers” were more abundant while the “stress-sensitives” and “mesophiles” were in lower proportion among the bacterial IT next to the oil seeps than in the control forest soil bacterial community (Fig. 5A). We previously described that competitors possessed traits improving the access to resources even when limited such as in oligotrophic environments and that Gram-positive and non-motile bacterial traits characterized this functional group. In contrast, colonizers were bacteria with high phenotypic plasticity with the ability to grow in anaerobic or facultative anaerobic conditions^39^. The higher proportion of bacteria belonging to these two functional groups among the IT could confirm the presence of two complementary populations for efficient hydrocarbon degradation next to the oil seeps.

There was also a higher proportion of known lithotrophs and of anaerobic or facultative anaerobic bacteria close to the oil seeps (Fig. 5A). Chemolithotropich bacteria close to the oil seeps could provide Fe(III) as electron acceptor to hydrocarbon-degraders^102^. Moreover, among bacterial IT, both aerobic and anaerobic/facultative anaerobic taxa were identified representing 62.5 and 33.1% respectively, while control bacterial community was mainly composed of aerobic bacteria. This finding agrees with higher rates of both aerobic and anaerobic hydrocarbon mineralization that were measured next to the oil seep (Table 2). These findings could indicate a zone of mixing between aerobic bacteria from surface soil and anaerobic bacteria brought to the surface soil with the oil seep as anaerobic hydrocarbon degraders prevail in sub-surface oil samples and contaminated petroleum groundwater^103,104^. The co-occurrence of different metabolisms has the advantage of reducing the concentrations of both toxic contaminants and of degradation metabolites^105^. Aerobic and anaerobic populations could alternatively be active depending on fluctuating redox conditions in the soil or could cooperate for hydrocarbon degradation such as in syntrophic metabolisms^21^ by substrates sharing^104,106^. Joint anaerobic and aerobic metabolisms were rarely suggested^107^, but recent evidence of the co-transcription of genes involved in both aerobic and anaerobic benzene degradation in continuous culture^108^ would explain our findings as well as previous ones of co-existence of aerobic and anaerobic bacteria in oil contaminated desert soils^55^.

At level 1 of KEGG functional categories, functions related to “Metabolism” and “Genetic information processing GIP” were detected in higher proportion while “Environmental information processing” and “Cellular processes” were detected in lower proportion for bacterial IT next to the oil seeps than for the control communities (Fig. 5B). Within the Metabolism category, “carbohydrate metabolism” and “nucleotide metabolism” were over-represented, with higher proportion of functions involved in glycolysis/gluconeogenesis, pentose phosphate pathway, and galactose, starch, sucrose, propanoate, C5-branched dibasic acid, and purine metabolisms. Some functions involved in the metabolism of lipids, cofactors and vitamins and terpenoids and polyketides were also detected in higher proportion. Similarly, Zhang et al. ^109^ showed that in sediment supplemented with pyrene, genes involved into almost every metabolic pathways showed a dramatic increase. Our finding indicates the possible availability of simple carbon sources besides hydrocarbons because these functions are dedicated to optimize the use of available resources and underline the large genetic investment of bacteria in the processing of environmental information important in these oil polluted environments^110^.

Moreover, within the “xenobiotic biodegradation and metabolism” category, functions involved in polycyclic aromatic hydrocarbon degradation as well as drug metabolism were significantly more present in bacterial IT increasing next to the oil seeps than in the control communities (Fig. 5B). All these traits indicate greater and different metabolic capacities of the bacterial IT identified, as well adapted to hydrocarbon contamination. Finally, within the GIP category, functions related to “Folding, sorting and degradation”, “Transcription” and “Translation” were also over-represented. These regulatory functions may be related to the adaptation of microorganism to this highly hydrocarbon-contaminated environment. Similarly, through genome analysis of the oil-degrading *Pseudomonas aeruginosa* N002 strain, authors found among other gene categories, a very high abundance of genes for transcription^111^.

## Conclusions

At this study site, where petroleum seeps have been contaminating the soil for at least a century, we were able to demonstrate the natural attenuation phenomenon. Indeed, the presence of microbial communities, that are well adapted to oil and its degradation, limits the extent of the contamination to a few meters around the seeps. We identified bacterial taxa that are well known for their capacity to biodegrade hydrocarbons, which it would be interesting to isolate to study their metabolism and use them for bioremediation purposes. Amongst the indicator taxa, which are highly favoured in the vicinity of oil seeps, we have shown the coexistence and possible cooperation of fungi as well as aerobic and anaerobic bacteria having specific functional traits that make them well adapted to the hydrocarbon contamination and to fluctuating redox conditions.

## Supplementary Information

Supplementary Information 1.

Supplementary Information 2.

Supplementary Information 3.

Supplementary Information 4.

Supplementary Information 5.
