# Author Correction: Taxonomic and functional trait-based approaches suggest that aerobic and anaerobic soil microorganisms allow the natural attenuation of oil from natural seeps

**DOI:** 10.1038/s41598-022-15752-z

**Published:** 2022-07-07

**Authors:** Aurélie Cébron, Adrien Borreca, Thierry Beguiristain, Coralie Biache, Pierre Faure

**Affiliations:** Université de Lorraine, CNRS, LIEC, 54000 Nancy, France

Correction to: *Scientific Reports* https://doi.org/10.1038/s41598-022-10850-4, Published online 04 May 2022

The original version of this Article contained an error in Table 2, where the data in column ‘Anaerobic mineralization CO_2_ production (µg C-CO_2_ g^−1^ d^−1^)’ was a duplication of the data of column ‘Aerobic mineralization CO_2_ production (µg C-CO_2_ g^−1^ d^−1^)’.

This error has now been corrected in the original version of the Article.

