# Supplementary material for: Taxonomic and functional trait-based approaches suggest that aerobic and anaerobic soil microorganisms allow the natural attenuation of oil from natural seeps
Source: Sci Rep. 2022 May 4;12:7245. doi: 10.1038/s41598-022-10850-4 (PMC9068923; doi:10.1038/s41598-022-10850-4)
Supplement: Supplementary file 1 — Supplementary Information 1. [file 41598_2022_10850_MOESM1_ESM.docx]

**Figure S1.** Fullscan chromatograms of the crude oils and the extractable organic matter (EOM) from samples taken at 0, 4 and 15 m along the transect of both Etangs-Verts (ETV) and Gunstett (GUN) sampling sites. Nomenclature of the identified compounds: *n*-C_x_ for *n*-alkanes, *n*-C_x_-Ol for alkanols, *n*-C_x_-Al for fatty aldehydes with x= carbon number.
