# Supplementary material for: Taxonomic and functional trait-based approaches suggest that aerobic and anaerobic soil microorganisms allow the natural attenuation of oil from natural seeps
Source: Sci Rep. 2022 May 4;12:7245. doi: 10.1038/s41598-022-10850-4 (PMC9068923; doi:10.1038/s41598-022-10850-4)
Supplement: Supplementary file 2 — Supplementary Information 2. [file 41598_2022_10850_MOESM2_ESM.docx]

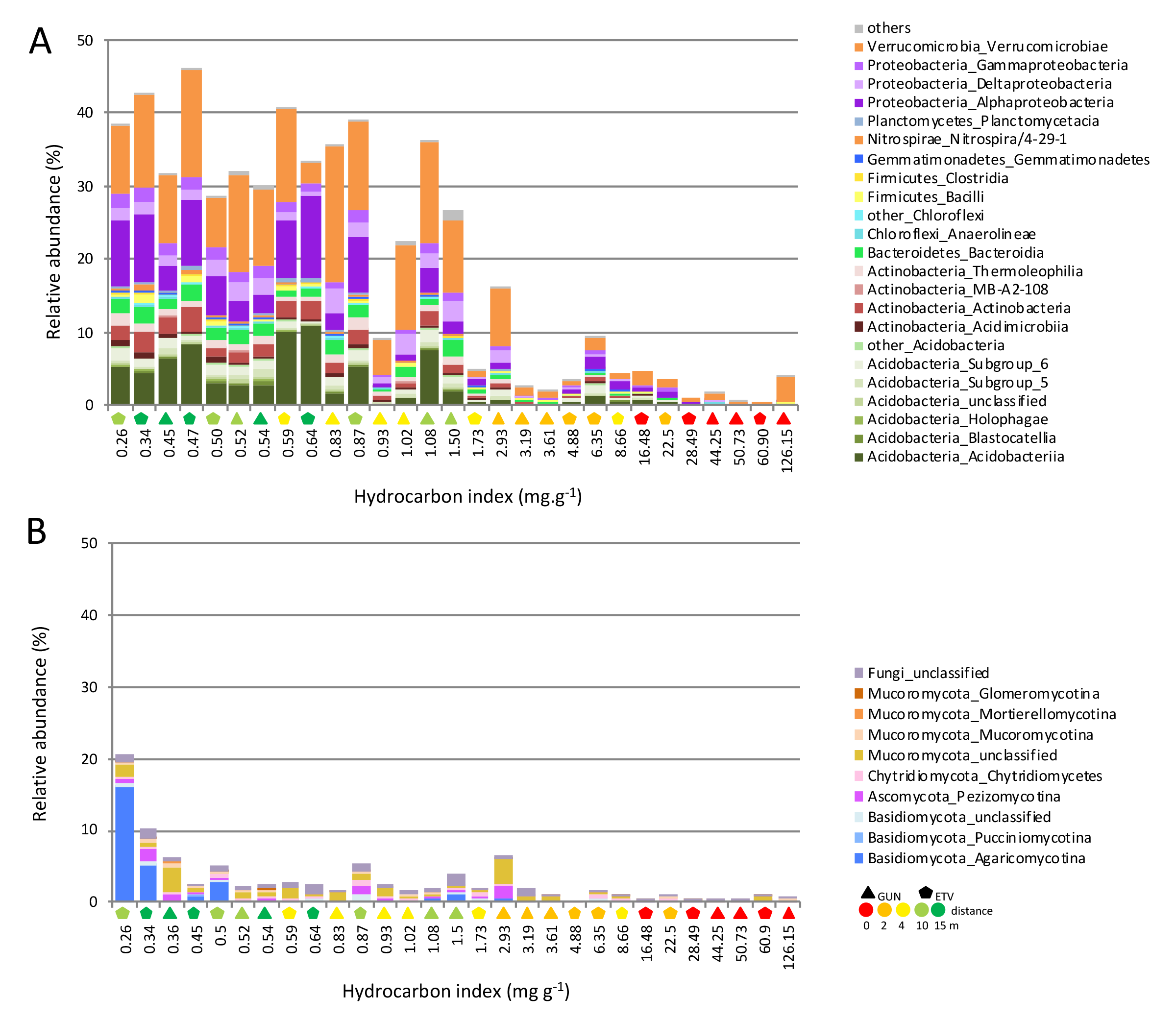


**Figure S2. Relative abundance of decreasing bacterial (A) and fungal (B) indicator taxa (summed at the class and sub-division level) for bacteria and fungi, respectively determined using TITAN2 along the gradient of petroleum contamination for the combination of both ETV and GUN sites.** *At the OTU level, 664 bacterial and 40 fungal decreasing indicator taxa were sorted with purity and reliability parameters both >0.95. For the complete list of bacterial and fungal OTU found as indicator taxa see* ***Table S2*** *and* ***S3****, respectively.*
